# The psychological impact of the COVID-19 pandemic on adults and children in the United Arab Emirates: a nationwide cross-sectional study

**DOI:** 10.1186/s12888-021-03213-2

**Published:** 2021-05-03

**Authors:** Basema Saddik, Amal Hussein, Ammar Albanna, Iffat Elbarazi, Arwa Al-Shujairi, Mohamad-Hani Temsah, Fatemeh Saheb Sharif-Askari, Emmanuel Stip, Qutayba Hamid, Rabih Halwani

**Affiliations:** 1 Department of Family and Community Medicine and Behavioral Sciences, College of Medicine, University of Sharjah, Sharjah, United Arab Emirates; 2 Sharjah Institute of Medical Research, University of Sharjah, Sharjah, United Arab Emirates; 3 Child and Adolescent Mental Health Center of Excellence, Al Jalila Children’s Specialty Hospital, Dubai, United Arab Emirates; 4 Mohamed Bin Rashid University of Medicine and Health Sciences, Dubai, United Arab Emirates; 5 Institute of Public Health, College of Medicine and Health Sciences, United Arab Emirates University, Al Ain, Abu Dhabi, United Arab Emirates; 6 Department of Pediatrics, College of Medicine, King Saud University, Riyadh, Kingdom of Saudi Arabia; 7 Prince Abdullah Ben Khaled Celiac Disease Research Chair, Department of Pediatrics, Faculty of Medicine, King Saud University, Riyadh, Kingdom of Saudi Arabia; 8 Department of Psychiatry and Behavioral Sciences, College of Medicine and Health Sciences, United Arab Emirates University, Al Ain, United Arab Emirates; 9 Department of Clinical Sciences, College of Medicine, University of Sharjah, Sharjah, United Arab Emirates

**Keywords:** COVID-19, Anxiety, Children, Adult, United Arab Emirates

## Abstract

**Background:**

The psychosocial impact of previous infectious disease outbreaks in adults has been well documented, however, there is limited information on the mental health impact of the COVID-19 pandemic on adults and children in the United Arab Emirate (UAE) community. The aim of this study was to explore anxiety levels among adults and children in the UAE and to identify potential risk and protective factors for well-being during the COVID-19 pandemic.

**Methods:**

Using a web-based cross-sectional survey we collected data from 2200 self-selected, assessed volunteers and their children. Demographic information, knowledge and beliefs about COVID-19, generalized anxiety disorder (GAD) using the (GAD-7) scale, emotional problems in children using the strengths and difficulties questionnaire (SDQ), worry and fear about COVID-19, coping mechanisms and general health information were collected. Descriptive analysis was carried out to summarize demographic and participant characteristics, Chi-square analysis to explore associations between categorical variables and anxiety levels and multivariable binary logistic regression analysis to determine predictors of anxiety levels in adults and emotional problems in children.

**Results:**

The overall prevalence of GAD in the general population was 71% with younger people (59.8%) and females (51.7%) reporting highest levels of anxiety. Parents who were teachers reported the highest percentage of emotional problems in children (26.7%). Adjusted multivariable logistic regression for GAD-7 scores showed that being female, high levels of worry associated with COVID-19, intention to take the COVID-19 vaccine and smoking were associated with higher levels of anxiety. Adjusted multivariable logistic regression for SDQ showed that higher emotional problems were reported for children in lower and higher secondary education, and parents who had severe anxiety were seven times more likely to report emotional problems in their children.

**Conclusions:**

This study reports the psychological impact of COVID-19 among adults and children in the UAE and highlights the significant association between parental and child anxiety. Findings suggest the urgency for policy makers to develop effective screening and coping strategies for parents and especially children.

**Supplementary Information:**

The online version contains supplementary material available at 10.1186/s12888-021-03213-2.

## Background

The coronavirus disease 2019 (COVID-19) emerged in Wuhan, China, December 2019, and was declared a public health emergency on January 30th 2020 [1] and a global pandemic by the World Health Organization (WHO) on March 11th [2]. By March 7th, 2021, 1 year after it was declared a pandemic, more than 117 million confirmed cases and almost 3 million deaths were reported worldwide, with 408,236 confirmed cases and 1310 deaths in the UAE [3]. In the absence of effective treatments and vaccines during the early stages of the pandemic, unprecedented public health interventions were implemented across the UAE to curb transmission of the disease. These included international border closures, travel bans, lockdowns, closures of schools and universities, strict social distancing, lockdowns and quarantines. These measures, along with fear of the pandemic and disruption in people’s lives have significant mental health implications [4].

Research on past infectious disease outbreaks, such as severe acute respiratory syndrome (SARS), swine flu, and influenza revealed a wide range of psychosocial impacts at individual, community, and international levels. These included worries about becoming infected and fear of dying [5], increase in anxiety, post-traumatic stress and depression [6], feelings of helplessness, guilt, panic and increased perception of risk [7–9]. More recently, studies investigating the psychological impacts of COVID-19 in China, Spain, Italy, India and the UK have reported moderate to severe stress, generalized anxiety, insomnia, and depression [10–16] associated with lockdowns, social isolation, changes in daily habits, public fear and worry.

Information about the mental health impact of COVID-19 on the UAE population is scarce. An earlier study explored the psychosocial correlates (COVID-19 infection status, mental health history, living arrangements and demographic variables) with depression and anxiety and reported high levels of anxiety and depression among segments of the UAE population [17]. However, no study has yet investigated correlates of anxiety with precautionary measures undertaken and lockdowns, coping mechanisms and perceptions of fear and worry. Furthermore, there are no published reports on the mental health impact of this pandemic on children. While severe COVID-19 is less frequent in children than in adults [18], the mental health of children may be disproportionately affected due to changes in their routines, school disruptions, reduction in social contact and fear of the unknown, all of which can cause heightened anxiety and impact on their well-being [19, 20].Previous pre-pandemic research from the UAE reports high levels of anxiety among adolescents [21] making this an especially vulnerable group to develop mental health problems because of the unique combination of the public health crisis, limited social contact, and schooling disruption [20]. Additionally, the impact of traumas and disasters on children’s mental health has been found to be influenced by the impact of post disaster traumas on parents, parenting, parent-child interactions, and the family environment [22]. With recent evidence on how parental anxiety can contribute to anxiety disorders in children [23–25] and the relationship between parental anxiety and child symptomatology [26, 27], the mental well-being of children during this pandemic should not be ignored. Parent and teacher observations are important in screening for psychological and emotional disorders in children and play a significant role in being key informants and data sources for measuring child psychosocial well-being [28, 29]. Furthermore, in times of paramount stress and uncertainty, parents and secure family environments are considered a safe haven for children and serve as strong protective factors against stress and anxiety. As observers and key informants, parents and teachers can positively influence children’s well-being [30, 31] .

In this study, we explored anxiety levels associated with the pandemic among adults and children in the UAE. We also examined the association between anxiety levels and demographics, knowledge, beliefs, hygienic practices, coping mechanisms, worry, fear and perceived risk related to COVID-19. This makes our study the first in the UAE to discuss this aspect in the current pandemic.

## Methods

### Participants

A stratified random sample of schools was selected from a list of schools in the UAE retrieved from the EdArabia website [32]. We randomly selected and contacted 17 schools to take part in the study. However, with school closures and the transition to online learning at the time of the study, only four schools responded and agreed to distribute the survey link with parents and teachers in their school. Using convenience and snowball sampling, participants were invited to take an online survey using email announcements through participating schools and posts on Facebook, Instagram and WhatsApp. Teachers, parents, and members of the public throughout the UAE, 18 years and older, participated and passed the survey link to friends. Data were collected from 24th March to 15th May, 2020. The survey was administered via the Survey Monkey platform [33], and each response came from a unique IP address to ensure unique entries. The first page explained the research objectives and assured confidentiality. The minimum sample size needed for this cross-sectional study was 385, calculated for an expected prevalence of 50%, margin of error of 5, and 95% confidence.

### Ethical approval and consent

The study was approved by the University of Sharjah Ethics Committee (approval number REC-20-03-12-01) and the United Arab Emirates University research ethics review board (ERS_2020_6098) and all research was performed in accordance with regulations of these committees. Participants gave online written consent to participate in the study prior to starting the survey.

### Data collection

A structured questionnaire comprising 32 items was used. Questions were divided into eight domains: demographics, knowledge, beliefs and perceived risk related to COVID-19, health-protective and hygienic behaviors, precautionary measures, worry and fear associated with COVID-19, general health, validated self-reported anxiety screening scales (adults and children) and coping mechanisms. The questionnaire (Appendix 1) was translated into Arabic by a certified translator, and back-translated to English to ensure accuracy. The final version was piloted among ten members of the general community to ensure clarity and consistency. The questionnaire was sent to a group of ten experts consisting of faculty, teachers, parents, and a mental health expert who reviewed the survey for accuracy, length, clarity and comprehensiveness. Modifications were made to questions and response items based on expert recommendations. The questionnaire took ten minutes to complete.

#### Demographics

Information was collected on participants’ age, sex, educational level, emirate or country of residence, marital status, number of children, ages and level of schooling, employment status, monthly income and health insurance. Participants indicated if they were parents, a parent and teacher, teachers only or neither parent nor teacher.

#### Knowledge, beliefs and perceived risks related to COVID-19

Participants were asked to answer “true”, “false”, or “don’t know” on statements related to COVID-19, such as “*there is no specific treatment*” and “*I feel a sense of social responsibility by staying at home*”. Perceived risk from COVID-19 was assessed on a 4-point Likert scale (very likely to not likely at all) where participants responded to the likelihood of contracting COVID-19, surviving COVID-19 or developing severe illness.

#### Health-protective practices and hygienic behaviors

Participants described how often they followed hygienic measures. Responses to seven questions (covering mouth when sneezing/coughing, using hand sanitizer, hand-washing, wearing facemasks, avoiding crowded areas, public transport and handshakes) were measured on a 4-point Likert scale (always to never). These questions were modified from versions used in studies during MERS-CoV, swine flu and SARS [7, 9, 34, 35]. To categorize hygienic behavior into dichotomous types, a standard median split was performed [36] with a median cut-off of 25. A value of ≥25 indicated high exhibiting behaviors.

#### Worry and fear associated with COVID-19

To assess worry and fear of COVID-19, participants were asked to rate how worried they were on seven questions: *Worried about catching COVID-19 myself; Worried about parents catching COVID-19; Worried about child catching COVID-19; Worried about what COVID-19 can do to me health-wise; Worried about social isolation/quarantine; Worried about loss of income; and Worried about transmitting the virus to family and friends*. Participants responded on a 5-point Likert scale from extremely worried to not worried at all. Participants were also asked their opinion on public fear associated with COVID-19 on a 5-point Likert scale (strongly agree to strongly disagree) [37]. To categorize worry into dichotomous categories, a standard median split was carried out [36] with a median cutoff of 22. A value of ≥22 was signified as very worried.

#### Anxiety

Anxiety among adults was measured using the generalized anxiety disorder scale (GAD-7) [38] which is a self-reported 7-item validated scale. Participants indicated how often they were bothered during the previous 2 weeks by symptoms of *feeling nervous, not being able to stop worrying, worrying about different things, trouble relaxing, restless, irritable and afraid that something awful might happen*. Response options were “not at all,” “several days,” “more than half the days,” and “nearly every day,” scored as 0, 1, 2, and 3. A score of ≥10 identified cases of anxiety with 89% sensitivity and 82% specificity, good internal consistency (Cronbach α = .92) and test-retest reliability (intraclass correlation = 0.83) [38]. Other research established a cutoff of 8, (sensitivity 77%, specificity 82%) as a screener for panic disorder, social anxiety phobia and PTSD [39]. GAD-7 scores were totaled and classified as minimal (0–4), mild (5–9), moderate (10–14) and severe (15–21) [38], and stratified into two groups (< 8 or > 8) as a cut-off for panic disorder and social anxiety phobia [39].

Children’s anxiety levels were measured using the emotional symptoms sub-scale from the strengths and difficulties questionnaire (SDQ) [40], which covers emotional symptoms, conduct problems, hyperactivity-inattention, peer relationship problems, and prosocial behaviors. It was designed to screen for psychological disorders in children aged 3 to 16 years [29]. The emotional symptoms sub-scale [41] asks parents and teachers questions about symptoms they have witnessed in children: *Often complains of headaches, stomach-ache or sickness; Many worries, often seems worried; Often unhappy, down-hearted or tearful; Nervous or clingy in new situations, easily loses confidence;* and*, Many fears, easily scared*. Each item can be marked “not true” (0), “somewhat true” (1) or “certainly true” (2) thereby generating a score of 0–10. A cutoff score of 7 indicates generalized anxiety disorder (sensitivity 75%, specificity 80%) and depressive and generalized anxiety disorders (sensitivity 67%, specificity 81%) [42]. According to scoring guidelines [43], an abnormal emotional problems score completed by both parents and teachers ranged from 5 to 10 with SDQ ≥5 indicating abnormal emotional problems and a score of 4 indicating borderline problems Validated Arabic translations of both the GAD-7 and SDQ were used for the Arabic translation of the questionnaire [44, 45].

To determine the impact of measures to reduce anxiety, participants were asked whether they felt less anxious with the introduction of *online learning, airport screening, travel bans, availability of hand sanitizer in public places, cancellation of social events, temporary closure of public places and social isolation*. Responses were recorded on a 5-point Likert scale (strongly agree to strongly disagree). To categorize precautionary measures into dichotomous categories, a standard median split was carried out [36] with a median cutoff of 34. A score of ≥34 indicated high agreement with precautionary measures.

#### Coping mechanisms

Participants were asked to indicate on a 4-point Likert scale (always to never) which coping mechanisms they used to reduce anxiety in their children and family. Questions included: *openly discussing COVID-19 with children/family, educating children about proper hygiene, assuring children they are safe, limiting children’s exposure to news coverage and social media, creating a schedule of learning and fun activities and maintaining regular routine*. To categorize coping strategies into dichotomous categories a standard median split was carried out [36] with a median cutoff of 18. A score of ≥18 indicated high coping strategies.

#### General health

Participants were asked whether they suffered from chronic disease or had flu-like symptoms over the previous 2 weeks, the treatments for such symptoms, the likelihood of taking a COVID-19 vaccine, whether their children were vaccinated, whether they smoked and if their smoking habits had changed since the outbreak.

### Statistical analysis

Descriptive statistics, including means, medians, frequencies and percentages were used to summarize data and to illustrate participants’ demographics and characteristics. The normal distribution of data was verified visually using histograms, boxplots, and quantile-quantile plots, and statistically using the Kolmogorov-Smirnov test. The equality of variances was checked using Levene’s test. Chi-square (χ2) tests explored associations between participant demographics, knowledge, health protective practices and hygienic behavior, general health, worry and fear, coping mechanisms, and anxiety levels. Statistically significant factors in the chi-square analysis were included in multivariable binary logistic regression models to determine predictors of anxiety levels (GAD-7 score ≥ 8) and emotional problems in children (SDQ score ≥ 5). The automatic selection of predictors in the model was performed by a stepwise backward method with an entry threshold of 0.05 and an exit threshold of 0.1. The adequacy of the models was verified by the Hosmer and Lemeshow test and the specificity of the model by Link Test. The estimates of the strengths of associations were demonstrated by the odds ratio (OR) with a 95% confidence interval (CI). A two-tailed *p < 0.05* was considered statistically significant. Data were analyzed using statistical software SAS® 9.3 [46].

## Results

In total, 2200 people completed the online participant sheet and consent form. Of these, 26 indicated they did not wish to proceed further and 381 completed only the demographic part of the questionnaire before discontinuing. Complete data were analyzed for 1469 participants (68%). Table 1 summarizes the demographics. Participants were primarily female (82.8%), 25 to 44 years of age (61.7%) and resided in the UAE (72.8%). Over half of our population held a bachelor’s degree (50.8%) and were employed (63.1%). Seventy five percent of participants were married and had children (75.6%), with the majority having 1–2 children (35.2%). The most commonly reported medical conditions were high blood pressure (9.1%) and asthma (8.6%). Headaches (25.9%) were the most commonly reported COVID-19 symptom and almost half of participants indicated they used vitamin C to treat their symptoms. Whilst most participants reported they did not smoke, 13.7% stated they had changed their smoking habits since the COVID-19 outbreak. Most indicated they would get vaccinated (71.5%) and have their children vaccinated (59.4%) against COVID-19. The majority indicated their children were current with vaccinations (85%); however, we found a significant association between those who reported their children were not current with vaccinations (53%) and their intention to not vaccinate their children against COVID-19.

**Table 1:** Demographic characteristics by anxiety score (GAD-7 ≥ 8) and children emotional SDQ score (SDQ ≥ 5) *(N = 1469)*

	Demographics			Anxiety GAD-7 Score (≥8)			Reported Children Emotional SDQ Score (SDQ ≥ 5)		
Variable	Category	Frequency (n)	%	%(n)	Chi- square χ2	***p-value***	%(n)	Chi-square χ2	***p-value***
Sex	Female	1216	82.8	51.7 (629)	10.16	0.001^*^	16.2 (154)	2.83	0.093
	Male	253	17.2	40.7 (103)			11.5 (24)		
Relationship to child/ren	Parent	893	60.8	48.2 (430)	4.24	0.120	14.6 (130)	25.6	< 0.001^*^
	Teacher only	106	7.2	44.3 (47)			4.7 (5)		
	Parent & teacher	161	11.0	55.9 (90)			26.7 (43)		
	No children	299	20.4	54.2 (162)			0		
Age	18–24	169	11.5	59.8 (101)	22.27	< 0.001^*^	8.7 (2)	3.44	0.329
	25–44	907	61.7	51.0 (463)			14.7 (115)		
	45–64	381	25.9	43.6 (166)			17.4 (61)		
	65+	12	0.8	16.7 (2)			0		
Education	Primary	11	0.7	36.4 (4)	3.14	0.534	11.1 (1)	1.76	0.779
	Lower secondary	10	0.7	30.0 (3)			25.0 (2)		
	Higher secondary	137	9.3	53.3 (73)			18.2 (18)		
	Bachelor degree	746	50.8	50.1 (374)			14.4 (79)		
	Post-graduate	565	38.5	49.2 (278)			15.8 (78)		
Country of Residence	Outside the UAE	400	27.2	49.0 (196)	0.15	0.697	15.6 (51)	0.02	0.882
	Inside the UAE	1069	72.8	50.1 (536)			15.2 (127)		
Employment	Employed	927	63.1	48.8 (452)	2.00	0.372	15.6 (122)	1.08	0.583
	Not employed	319	21.7	53.3 (170)			12.9 (22)		
	Home duties	223	15.2	49.3 (110)			16.6 (34)		
Monthly Salary	Other	192	13.1	46.4 (89)	5.19	0.393	15.2 (25)	9.90	0.079
	less than 5000	161	11.0	55.9 (90)			12.4 (13)		
	5000-9999	163	11.1	54.0 (88)			20.2 (23)		
	10,000-19,999	282	19.2	48.9 (138)			15.8 (35)		
	20,000-39,000	413	28.1	49.6 (205)			18.1 (60)		
	40,000+	258	17.6	47.3 (122)			9.9 (22)		
Insurance	Other	21	1.4	47.6 (10)	0.634	0.729	15.4 (2)	4.13	0.126
	No	226	15.4	52.2 (118)			10.1 (17)		
	Yes	1222	83.2	49.4 (604)			16.2 (159)		
Marital Status	Single	287	19.5	55.7 (160)	7.27	0.064	4.2 (3)	11.9	0.008^*^
	Married	1094	74.5	47.8 (523)			15.5 (157)		
	Divorced/ Separated	72	4.9	56.9 (41)			25.4 (16)		
	Widowed	16	1.1	50.0 (8)			14.3 (2)		
Do you have children?	Yes	1111	75.6	49.1 (545)	1.10	0.296			
	No	358	24.4	52.2 (187)					
Number of children	1–2 children	517	35.2	49.7 (257)	4.85	0.089	16.6(80)	0.070	0.966
	3–4 children	473	32.2	50.5 (239)			16.0(73)		
	4+ children	119	8.1	39.5 (47)			16.5(19)		
Age category of children	Infants and Toddlers	269	18.3	52.4 (141)	0.88	0.348	16.2 (154)	0.167	0.683
	Preschoolers	325	22.1	53.8 (175)	2.69	0.101	11.5 (24)	0.085	0.770
	School Age	713	48.5	50.5 (360)	0.24	0.623	14.6 (130)	5.93	0.015^*^
	Adolescents	432	29.4	48.6 (210)	0.36	0.547	17.5 (121)	8.83	0.003^*^
	Young Adults	254	17.3	42.5 (108)	6.56	0.010^*^	19.6 (81)	1.53	0.216
Children attending school	Children don’t go to school	183	12.5	55.7 (102)	2.92	0.088	13.6(22)	0.45	0.502
	Childcare	205	14.0	50.2 (103)	0.01	0.898	16.4 (32)	0.20	0.651
	Primary	668	45.5	52.4 (350)	3.23	0.073	16.8 (109)	2.39	0.123
	Lower secondary	400	27.2	49 (196)	0.15	0.697	21.4 (82)	16.2	< 0.001^*^
	Higher Secondary	320	21.8	45.6 (146)	2.89	0.089	20.5 (63)	8.61	0.003^*^
	University	258	17.6	42.6 (110)	6.48	0.011^*^	17.4 (40)	0.92	0.336
Likely to vaccinate self	No	383	26.1	41.3 (158)	20.05	< 0.001^*^			
	Yes	1050	71.5	53.2 (559)					
Likely to vaccinate children	No	314	21.4	41.7 (131)	20.71	0.001^*^			
	Yes	872	59.4	52.4 (457)					
Do you have any of the following medical Conditions (Yes)	Diabetes	75	5.1	53.3 (40)	0.39	0.533			
	Heart Problems	36	2.5	58.3 (21)	1.07	0.301			
	High Blood Pressure	133	9.1	51.9 (69)	0.25	0.620			
	Dyslipidemia	45	3.1	48.9 (22)	0.89	0.898			
	Asthma	127	8.6	62.2 (79)	8.52	0.004^*^			
	Respiratory problems	47	3.2	53.2 (25)	0.64	0.639			
	Cancer	9	0.6	44.4 (4)	0.75	0.746			
	Other medical conditions	128	8.7	50.8 (65)	0.82	0.822			
Have you experienced any of the following symptoms (Yes)	Headaches	381	25.9	56.7 (216)	9.69	0.002^*^			
	Fever	105	7.1	56.2 (59)	1.83	0.176			
	Cough	235	16.0	51.1 (120)	0.17	0.680			
	Difficulty breathing	37	2.5	51.4 (19)	0.03	0.851			
	Sore throat	256	17.4	59.8 (153)	12.2	< 0.001^*^			
	Myalgia	49	3.3	59.2 (29)	1.77	0.183			
	Dizziness	73	5.0	63 (46)	5.34	0.021^*^			
	Runny nose	225	15.3	53.8 (121)	1.66	0.198			
	Diarrhea	100	6.8	50 (50)	0.001	0.972			
	Other Symptoms	18	1.2	50 (9)	< 0.001	0.988			
What measures have you taken to treat your symptoms	Vitamin C	720	49.0	53.5 (385)	7.49	0.006^*^			
	Flu medications	92	6.3	53.3 (49)	0.46	0.497			
	Anti-inflammatory drugs	139	9.5	62.6 (87)	1.00	0.002^*^			
	Analgesics anti-pyretic	345	23.5	58.6 (202)	13.7	< 0.001^*^			
	Oral Steroids	17	1.2	58.8 (10)	0.55	0.456			
	Herbal remedies	443	30.2	51.2 (227)	0.51	0.477			
My child/ren are up to date with their vaccines	No	81	5.5	45.7 (37)	1.49	0.476			
	Yes	1029	70.0	49.5 (509)					
	I don’t have children	334	22.7	52.4 (175)					
Smoking	No	1196	81.4	47.3 (566)	19.01	< 0.001^*^			
	Yes	195	13.3	62.1 (121)					
	I used to smoke but quit	53	3.6	64.2 (34)					
Smoke type	Cigarettes	111	7.6	58.6 (65)	3.66	0.056			
	Shisha	83	5.7	65.1 (54)	8.16	0.004^*^			
	Midwakh	8	0.5	50 (4)	0.01	0.992			
	Vaping	22	1.5	59.1 (13)	0.76	0.381			
Smoking changed During COVID-19	Yes	201	13.7	58.7 (118)	7.73	0.005^*^			
	No	1228	83.6	48.1 (591)					
Changes made to smoking	Stopped/Decreased	73	5	54.4 (49)	2.80	0.422			
	Started/Increased	18	1.2	14.7 (13)					
**Anxiety (GAD-7) levels**	Minimal	447	30.4	–			3.9 (14)	122.19	< 0.001^*^
	Mild	465	32.7	–			9.6 (35)		
	Moderate	296	20.2	–			24.5 (58)		
	Severe	261	17.8	–			35.3 (71)		
**Difficulty getting things done**	Not difficult at all	450	30.6	16.2(73)	392.41	< 0.001^*^	4.5 (16)	738.01	< 0.001^*^
	Somewhat	747	50.9	55.3(413)			14.6 (87)		
	Very	186	12.7	88.7(165)			36.2 (55)		
	Extremely	86	5.9	94.2(81)			35.7 (20)		
**Anxiety GAD class(≥8)**	Mild-Minimal	737	50.2	–			25.4(144)	86.20	< 0.001^*^
	High	732	49.8	–			5.7 (34)		
**SDQ class (≥5)**	Normal	982	84.7	43.1(423)	86.3	< 0.001^*^			
	Abnormal	178	15.3	80.9(144)					

*Significant at *p* < 0.05

### Anxiety levels (GAD-7 score and SDQ score)

Almost three quarters (71%) of our adult population reported anxiety, and 38% had moderate to severe anxiety. When we categorized anxiety by high and low based on the GAD-7 cutoff of 8, half of our participants (49.8%) reported higher levels of anxiety. Females (51.7%) and participants between the ages of 18 and 24 years (59.8%) reported greater anxiety. Higher anxiety levels were reported amongst participants with higher levels of education, but differences were not significant. More than half of participants who indicated they were likely to be vaccinated against COVID-19 were more anxious. More than half of parents who indicated they were likely to vaccinate their children with the COVID-19 vaccine had higher anxiety levels. Higher levels of anxiety were reported by asthmatics and those who had experienced headaches, sore throat or dizziness. Highly anxious participants were more likely to take vitamin C (53.5%), anti-inflammatory drugs (62.6%) and analgesics (58.6%). Participants who had quit smoking had higher anxiety levels. (Table 1).

Parents reported abnormal emotional problems in just over 15% of children. If borderline SDQ scores are also taken into consideration, a quarter of children (24.6%) had reported emotional problems. The highest percentage of reported emotional problems for children was in participants who were both parents and teachers (26.7%) compared to parents only (14.6%) or teachers only (4.7%). Participants who were divorced/separated reported higher SDQ scores in their children (25.4%), compared to those who were married (15.5%) and school-aged children or adolescents showed significant differences in emotional problems compared to children who were not (17.5%) and (19.6%) respectively. Emotional problems were also more commonly reported among children attending lower secondary and higher secondary schools. Parents reporting moderate to severe anxiety levels in the GAD-7, also reported higher SDQ scores in their children. A higher percentage of parents of children with emotional problems also reported they found it “Very or extremely” difficult to get things done (36.1%) (Table 1).

### Knowledge, beliefs, hygienic behavior and anxiety

Overall, participants showed a good knowledge of COVID-19 and the majority were aware that there was no treatment. Participants (83%) perceived a likelihood of catching COVID-19 with almost half reporting higher levels of anxiety. More than half who believed they would develop severe illness upon contracting the virus reported higher levels of anxiety (Table 2). Almost all participants had made significant changes in their hygienic behavior since the pandemic and reported increased use of hand sanitizer (87%), washing hands (99%), wearing facemasks (47%), and avoiding crowds (96%), public transportation (98%) and handshaking (95%). Significantly higher levels of anxiety were reported amongst participants who always used hand sanitizers and face masks. When behavioral changes were further categorized into two groups, participants who always practiced hygienic behaviors, reported significantly higher levels of anxiety (Table 3).

**Table 2:** Prevalence of GAD-7 score ≥ 8 by knowledge and beliefs related to COVID-19 (*N* = 1469)

Characteristics	Category	Frequency (n)	%	Anxiety GAD-7 Score (≥8) % (n)	Chi Square χ2	***p-value***
**No Treatment Available for COVID-19**	Don’t know	187	12.7	54.5 (102)	2.68	0.262
	False	180	12.3	46.1 (83)		
	True	1102	75.0	49.6 (547)		
**I feel a Sense of Social Responsibility**	Don’t know	19	1.3	36.8 (7)	1.36	0.505
	False	15	1.0	46.7 (7)		
	True	1435	97.7	50 (718)		
**There is likelihood of catching COVID-19**	Don’t know	100	6.8	40.0 (40)	7.26	0.026^*^
	Not likely	155	10.6	43.9 (68)		
	Likely	1214	82.6	51.4 (624)		
**There is likelihood of surviving COVID-19**	Don’t know	113	7.7	54.0 (61)	3.14	0.208
	Not likely	50	3.4	60.0 (30)		
	Likely	1306	88.9	49.1 (64)		
**There is likelihood I will develop severe illness**	Don’t know	199	13.5	48.2 (96)	13.56	0.001^*^
	Not likely	433	29.5	43.0 (186)		
	Likely	837	57.0	49.8 (732)		

^*^Significant at *p* < 0.05

**Table 3:** Prevalence of GAD-7 score ≥ 8 by Hygiene behavior changes taken *(n = 1469)*

Characteristics	Category	Frequency (n)	%	Anxiety GAD-7 Score (≥8)% (n)	Chi-Square χ2	p-value^*^
**Cover mouth**	Never	13	0.9	46.2 (6)	3.03	0.219
	Occasionally	43	2.9	62.8 (27)		
	Most of the time /Always	1413	96.2	49.4 (699)		
**Use hand sanitizer**	Never	26	1.8	26.9 (7)	10.90	0.004^*^
	Occasionally	163	11.1	41.7 (68)		
	Most of the time /Always	1280	87.1	51.3 (657)		
**Washing hands**	Never	2	0.1	50.0 (1)	1.13	0.569
	Occasionally	14	1.0	35.7 (5)		
	Most of the time /Always	1453	98.9	50.0 (726)		
**Face mask**	Never	374	25.5	48.9 (183)	8.84	0.012^*^
	Occasionally	408	27.8	44.4 (181)		
	Most of the time /Always	687	46.8	53.6 (368)		
**Avoid crowds**	Never	6	0.4	50.0 (3)	0.25	0.882
	Occasionally	60	4.1	46.7 (28)		
	Most of the time /Always	1403	95.5	50 (701)		
**Avoid public transport**	Never	14	1.0	50.0 (7)	0.17	0.918
	Occasionally	22	1.5	45.5 (10)		
	Most of the time /Always	1433	97.5	49.9 (715)		
**Avoid handshaking**	Never	12	0.8	66.7 (8)	1.59	0.451
	Occasionally	62	4.2	46.8 (29)		
	Most of the time /Always	1395	94.9	49.8 (695)		
**Behavioral changes total Category**	Occasionally exhibiting behavior changes	604	41.1	45.4(274)	8.18	< 0.004^*^
	Always exhibiting behavior changes	865	58.9	53.0(458)		

^*^Significant at *p* < 0.05

### Precautionary measures and anxiety

Although most participants felt less anxious with the government’s precautionary measures, participants who disagreed reported higher GAD-7 scores for online learning, cancellation of social events and social isolation. Participants who agreed with overall precautionary measures showed significantly less anxiety than those who disagreed (Table 4).

**Table 4:** Prevalence of GAD-7 score ≥ 8 by opinions on precautionary measures taken *(N = 1469)*

Characteristics	Category	Frequency (n)	%	Anxiety GAD-7 Score (≥8) % (n)	Chi Square χ2	***p-value***
*I feel that my levels of anxiety have reduced with the introduction of the following precautionary measures*						
Online learning at educational institutions	Strongly disagree/Disagree	220	15.0	65.0 (143)	36.55	< 0.001^*^
	Neutral	238	16.2	57.6 (137)		
	Strongly agree/Agree	1011	68.8	44.7 (452)		
Airport screening	Strongly disagree/Disagree	67	4.6	61.2 (41)	14.14	0.001^*^
	Neutral	136	9.3	62.5 (85)		
	Strongly agree/ Agree	1266	86.2	47.9 (606)		
Travel bans	Strongly disagree/Disagree	65	4.4	55.4 (36)	11.11	0.004^*^
	Neutral	79	5.4	67.1 (53)		
	Strongly agree/Agree	1325	90.2	48.5 (643)		
Hand sanitizers in public spaces	Strongly disagree/ Disagree	33	2.2	51.5 (17)	2.31	0.315
	Neutral	98	6.7	57.1 (56)		
	Strongly agree/ Agree	1338	91.1	49.3 (659)		
Cancellation of social events	Strongly disagree/Disagree	34	2.3	61.8 (21)	7.59	0.022^*^
	Neutral	50	3.4	66.0 (33)		
	Strongly agree /Agree	1385	94.3	49.0 (678)		
Temporary closure of public places	Strongly disagree/Disagree	47	3.2	51.1 (24)	1.76	0.415
	Neutral	51	3.5	58.8 (30)		
	Strongly agree/Agree	1371	93.3	49.5 (678)		
Social isolation	Strongly disagree /Disagree	49	3.3	61.2 (30)	7.26	0.026^*^
	Neutral	82	5.6	61.0 (50)		
	Strongly agree/Agree	1338	91.1	48.7 (652)		
**Precautionary measures category**	Disagree with precautionary measures	605	41.2	54.9 (332)	10.48	0.001^*^
	Agree with precautionary measures	864	58.8	46.3(400)		

^*^Significant at *p* < 0.05

### Worry, fear and anxiety

The majority of participants felt public fear was justified. However, we found greater anxiety among those who believed that fear caused unnecessary absences from work and school. Whilst most participants worried about contracting COVID-19, the majority were more worried about their parents (75%) or children (65.5%) catching COVID-19 or transmitting it to someone else if they caught it (64.5%). Significantly higher GAD-7 scores were found among all participants who agreed they were worried about catching COVID-19, their parents or children catching it, about what would happen if they caught it, about being in social isolation, loss of income and transmitting it to others. When we categorized worry into two groups, “low levels of worry” and “high levels of worry”, we found significantly higher levels of anxiety among participants who reported being very worried (Table 5). Worry in parents was associated with SDQ score, and parents with higher scores reported more emotional problems in their children. Parents who were very worried reported significantly higher SDQ scores for their children (Table 5).

**Table 5:** Worry about COVID-19 by GAD-7 score ≥ 8 and reported Child SDQ score ≥ 5 *(N = 1469)*

				Anxiety GAD-7 Score (≥8)			Reported Children Emotional SDQ Score (SDQ ≥ 5)		
Characteristics	Category	Frequency (n)	%	%(n)	Chi-Square χ2	p-value	%(n)	Chi-square χ2	***p***-value
I believe the public fear is justifiable	Strongly disagree/Disagree	85	5.8	31.8 (27)	29.08	< 0.001^*^	29.7 (11)	0.89	0.640
	Neutral	156	10.6	35.3 (55)			9.5(71)		
	Strongly agree /Agree	1228	83.6	52.9 (650)			29.2 (186)		
I believe the public fear is dysfunctional	Strongly disagree/Disagree	759	51.7	51.5 (391)	10.86	0.004^*^	28.5 (107)	0.357	0.836
	Neutral	261	17.8	40.6 (106)			13.9(29)		
	Strongly agree/ Agree	448	30.6	52.3 (235)			30 (74)		
I am worried about catching COVID-19	Not worried at all	138	9.5	21.7 (30)	176.98	< 0.001^*^	6.6 (7)	55.25	≤0.001^*^
	Little/Somewhat worried	801	55.2	40.6 (325)			10.3 (67)		
	Very/Extremely worried	511	35.2	72.6 (371)			26.3 (104)		
I am worried about my parents catch COVID-19	Not worried at all	49	3.6	14.3 (7)	98.86	≤0.001^*^	5.1 (2)	18.24	≤0.001^*^
	Little/Somewhat worried	294	21.4	29.6 (87)			8.2(20)		
	Very/Extremely worried	1028	75.0	57.8(594)			18.4 (146)		
I am worried my children catch COVID-19	Not worried at all	62	5.3	19.4 (12)	110.44	≤0.001^*^	4 (2)	23.14	≤0.001^*^
	Little/Somewhat worried	342	29.2	29.8(102)			10 (32)		
	Very/Extremely worried	766	65.5	60.2(461)			20.4 (141)		
I am worried about what COVID-19 can do to me health wise	Not worried at all	121	8.4	17.4 (21)	178.81	≤0.001^*^	3.1 (3)	44.22	≤0.001^*^
	Little/Somewhat worried	667	46.4	38.4 (256)			10.5 (56)		
	Very/Extremely worried	647	45.1	68.8 (445)			23.1 (117)		
I am worried about social isolation	Not worried at all	375	26.1	34.4(129)	81.71	≤0.001^*^	9.2 (27)	58.29	≤0.001^*^
	Little/Somewhat worried	608	42.3	48.2(293)			10.6 (52)		
	Very/Extremely worried	454	31.6	65.6(298)			27.7 (99)		
I am worried about loss of income if infected with COVID-19	Not worried at all	265	18.8	32.1 (85)	73.63	≤0.001^*^	7.9 (16)	22.91	≤0.001^*^
	Little/Somewhat worried	441	32.9	44.4 (196)			12.1 (44)		
	Very/Extremely worried	633	47.3	61.6 (390)			20.7(107)		
I am worried I transmit COVID-19 to others	Not worried at all	104	8.5	31.7 (33)	79.81	≤0.001^*^	8.5 (8)	32.48	≤0.001^*^
	Little/Somewhat worried	328	27.0	34.5 (113)			7.4 (20)		
	Very/Extremely worried	785	64.5	60.5 (475)			21.6 (131)		
**Overall worry about COVID-19**	Low levels of worry	394	26.8	27.4 (108)	148.7	≤0.001^*^	6.4 (18)	44.9	≤0.001^*^
	High levels of worry	1075	73.2	58 (624)			18.2 (160)		

^*^Significant at *p* < 0.05

Among participants with children, most were utilizing effective coping strategies; however, higher anxiety was reported among participants who always openly discussed COVID-19 with their family (51.4%), compared to those who never did (33.3%). Participants who always educated their children about proper protective measures (50.3%) or limited news exposure (53.4%) had higher anxiety levels compared to those who never did these things (23.1%) and (41%) respectively. When we categorized these strategies into two groups low use and high use of coping strategies, we found no differences in anxiety levels based on GAD-7 score. For SDQ scores reported by parents, we found more emotional problems in children whose parents/teachers discussed COVID-19 with them (17.5%) and among those who educated their children about personal protective measures (20.9%). Parents who always utilized coping strategies for dealing with COVID-19, reported greater emotional problems in their children than parents who used fewer coping strategies (Table 6).

**Table 6:** Coping strategies used with children during COVID-19 by GAD-7 score and SDQ score (N = 1469)

				Anxiety GAD-7 Score (≥8)			SDQ Score (SDQ ≥ 5)		
Characteristics	Category	Frequency (n)	%	%(n)	Chi-square χ2	***p-value***	%(n)	Chi-square χ2	***p-value***
I have openly discussed COVID-19 with my family	No children	178	12.1	53.9 (96)	10.79	0.013^*^	2.7 (2)	13.42	0.004^*^
	Never	27	1.8	33.3 (9)			4.2 (1)		
	Occasionally	259	17.6	42.5 (110)			14.1 (30)		
	Most of the time/Always	1005	68.4	51.4 (517)			17.1 (145)		
I have educated my children about PPE	No children	316	21.5	52.2 (165)	10.19	0.017^*^	2.4 (3)	21.27	≤0.001^*^
	Never	13	0.9	23.1 (3)			0 (0)		
	Occasionally	56	3.8	33.9 (19)			10.9 (5)		
	Most of the time/Always	1084	73.8	50.3 (545)			17.3 (170)		
I reassure my children they are safe	No children	331	22.5	52.6 (174)	1.89	0.595	2.3 (3)	23.80	≤0.001^*^
	Never	20	1.4	45 (9)			0 (0)		
	Occasionally	92	6.3	45.7 (42)			20.3 (16)		
	Most of the time/Always	1026	69.8	49.4 (507)			17 (159)		
I have limited news exposure	No children	407	27.7	50.9 (207)	11.20	0.011^*^	3 (6)	37.10	≤0.001^*^
	Never	205	14.0	41 (84)			11.2 (21)		
	Occasionally	216	14.7	45.8 (99)			22.1 (43)		
	Most of the time/Always	641	43.6	53.4 (342)			18.6 (108)		
I have created a schedule for learning	No children	335	22.8	48.4 (162)	0.51	0.916	6.6 (10)	10.40	0.015^*^
	Never	104	7.1	51 (53)			17.4 (16)		
	Occasionally	288	19.6	51 (147)			16.4 (42)		
	Most of the time/Always	742	50.5	49.9 (370)			16.7 (111)		
I have maintained a regular routine	No children	225	15.3	50.7 (114)	2.26	0.521	4.4 (4)	8.99	0.029^*^
	Never	47	3.2	46.8 (22)			14.7 (5)		
	Occasionally	185	12.6	54.6 (101)			16.2 (25)		
	Most of the time/Always	1012	68.9	48.9 (495)			16.3 (144)		
**Overall Coping strategies Total Category**	Low use of coping strategies	705	48.0	49.7(366)	1.65	0.199	5.6 (10)	9.01	< 0.001^*^
	High use of coping strategies	765	52.0	50.3(371)			17.1 (168)		

^*^Significant at *p* < 0.05

To estimate the probability of anxiety levels among participants in our study, two multivariable logistic regressions were conducted--one with the GAD-7 score ≥ 8 as a measure of adult anxiety and the other with the SDQ score ≥ 5 for anxiety and emotional problems in children. In the first model, the effects of adults sex, adults age, age of children, adults perception of fear, perception of likelihood to contract COVID-19 and to develop severe disease, headaches, sore throat, asthma, measures taken for symptoms, smoking, and changed smoking habits, likelihood of vaccination for self and children, hygienic behavior category, precautionary measures category and worry category were modelled. The omnibus model for logistic regression analysis was statistically significant χ^2^ (40, *N* = 1469) = 276.2, *p* ≤ 0.001. The model explained 28% (Nagelkerke R^2^) of the variance in anxiety levels. Hosmer and Lemeshow test results confirmed the model was a good fit for the data χ^2^(8, N = 1469) = 7.16, *p* = 0.519 (Table 7). Females had 1.91 times higher odds of reporting anxiety than males, and participants who believed that fear was justified were six times more anxious than those who disagreed. Higher levels of worry were also associated with increased anxiety levels. Participants who said they would take the COVID-19 vaccine were 1.57 times more likely to report higher anxiety, however, likelihood to vaccinate children did not influence anxiety (*p* = 0.158). The odds of higher anxiety were larger among participants who smoked, took vitamin C for symptoms and reported sore throat (Table 7).

**Table 7:** Predictors for anxiety (GAD-7 score ≥ 8) in adult population and predictors for parent/teacher reported emotional problems in children (SDQ score ≥ 5) using multivariable logistic regression analysis

Variable		***b***	***SE(b)***	***P-value***	***aOR [95% CI]***
***Generalized Anxiety Disorder (GAD-7) score (n = 1469)#***					
**Sex**	Female	0.649	0.178	< 0.001	1.91 [1.35–2.71]
	Male^a^	–	–	–	1
**Precautionary Measures**	Agree	−0.740	0.146	< 0.001	0.48 [0.36–0.63]
	Disagree^a^	–	–	–	1
**Public fear Justifiable**	Agree	1.811	1.082	0.094	6.11 [0.73–51.0]
	Disagree^a^	–	–	–	1
**Levels of Worry associated with COVID-19**	High	1.336	0.139	< 0.001	3.80 [2.90–5.00]
	Low^a^	–	–	–	1
**Will take COVID-19 Vaccine**	Yes	0.446	0.1478	0.003	1.57 [1.17–2.09]
	No^a^	–	–	–	1
**Symptoms- Sore throat**	Yes	0.447	0.173	0.010	1.56 [1.17–2.09]
	No^a^	–	–	–	1
**Taking Vitamin C**	Yes	0.344	0.134	0.010	1.41 [1.09–1.83]
	No^a^	–	–	–	1
**Smoker**	Yes	0.435	0.194	0.025	1.55 [1.06–2.26]
	No^a^	–	–	–	1
Model fit: Hosmer and Lemeshow test χ^2^(8, N = 1469) = 7.16, *p* = 0.519–2 log likelihood 1081.692					
Strengths and Difficulties Questionnaire (SDQ) score (n = 1160)*					
**Adult relationship to child/ren**	Parent only	0.854	0.493	0.884	2.35 [0.89–6.17]
	Parent & Teacher	1.626	0.519	< 0.001	5.08 [1.84–14.0]
	Teacher only^a^	–	–	–	1
**Child/ren in lower secondary education**	Yes	0.522	0.189	0.006	1.69 [1.16–2.44]
	No^a^	–	–	–	1
**Child/ren in higher secondary education**	Yes	0.460	0.199	0.021	1.59 [1.07–2.34]
	No^a^	–	–	–	1
**Anxiety level (GAD-7)**	Severe	1.94	0.355	< 0.001	7.00 [3.45–14.0]
	Moderate	1.505	0.340	0.013	4.51 [2.31–8.80]
	Mild	0.582	0.344	0.011	1.79 [0.91–3.50]
	Minimal^a^	–	–	–	1
**Difficulty of parent/teacher to get things done**	Extremely	1.299	0.439	0.003	3.70 [1.55–8.66]
	Very	1.403	0.348	< 0.001	4.07 [2.10–8.05]
	Somewhat	0.805	0.306	0.009	2.24 [1.23–4.08]
	Not difficult at all^a^	–	–	–	1
Model fit: Hosmer and Lemeshow test χ^2^ (7, N = 1160) =11.99, *p* = 0.101); −2 log likelihood 764.550					

^a^ reference group,* b* parameter estimate, *SE* Std Error, *aOR* Adjusted Odds Ratio, *CI* Confidence Interval. ^#^Logistic regression adjusted for the effects of sex, age, age of children, perception of fear, perception of likelihood to contract COVID-19 and to develop severe disease, headaches, sore throat, asthma, measures taken for symptoms, smoking, and changed smoking habits, likelihood of vaccination for self and children, hygienic behavior and attitudes towards precautionary measures

^*^Logistic regression adjusted for adult’s relationship to child, age (school-aged or adolescent), marital status, educational level of child (lower secondary and higher secondary), coping strategies, worry, parental anxiety levels (GAD-7) and difficulty getting things done

In the second model, with SDQ ≥ 5 as a measure of anxiety in children, the effects of relationship of the adult completing the survey to the child, adult’s marital status, child’s age (school-aged or adolescent),, educational level of child (lower secondary and higher secondary), parental coping strategies, worry, parental anxiety level and parental reports of difficulty getting things done were modelled. The omnibus model for logistic regression analysis was significant χ^2^ (17, *N* = 1160) =185.90, *p* ≤ 0.001 and explained 26% (Nagelkerke R^2^) of the variance in children’s anxiety levels. Hosmer and Lemeshow test results confirmed the model was a good fit χ^2^ (7, N = 1160) =11.99, *p* = 0.101 (Table 7). Participants who were both parents and teachers were five times more likely to report emotional problems in children mostly in adolescents in lower and secondary school. Parents with severe anxiety levels were seven times more likely to report emotional problems in their children. Parental reports of “finding it very difficult to do work, to do things at home and to get along with other people” were a strong predictor of emotional problems in children (Table 7).

## Discussion

This study revealed that the pandemic has had a significant impact on the mental health and well-being of the UAE population with the majority of adult participants reporting moderate to severe anxiety. This was most prevalent among women which is consistent with other research showing higher prevalence of anxiety among females compared to males [47–49]. Female anxiety during COVID-19 may be exacerbated by socio-cultural norms and gender-role expectations particularly with the added responsibility of home schooling, work commitments, social isolation and increased concern for family and loved ones. We also found that government measures to contain the virus were correlated with lower levels of anxiety. However, higher levels of anxiety were reported among those who had concerns about online learning which could be due to the disruption caused in their children’s education and examinations. Airport closures, screenings and travel bans were also found to be major triggers for anxiety which could be explained by the UAE being a popular travel hub and home to over seven million expatriates. The potential loss of jobs, financial insecurity, suspension of work visas, inability to travel to family and loved ones and overall loss of connection with the world are significant causes of worry [50–52]. Greater worry in our study was correlated with higher GAD-7 scores for concerns over parents’ and children’s health, fears of bringing infection home from the workplace and loss of income if infected with COVID-19. Parental levels of worry were associated with emotional problems in children, but further analysis showed lower correlation.

Perceptions of greater risk corresponded to increased anxiety. Participants in our study perceived a high risk of COVID-19 contagion and if infected, they perceived high risk of developing severe disease. These findings contradicted research conducted in China during the early stages of the pandemic where participants reported lower perceived likelihood of contracting COVID-19, which was associated with lower stress [11]. High-risk perception among participants in our study could also explain the high compliance of protective and hygienic behaviors such as handwashing and social distancing. Earlier research indicates that people who were more anxious about contracting COVID-19 were also more engaged in regular hand hygiene and social distancing behaviors [53, 54]. In our study, the majority who had higher GAD-7 scores reported wearing masks and using hand sanitizers. Pre-existing health conditions also create a sense of panic and concern. As demonstrated in our study, those with health conditions like asthma were more likely to feel concern because of probability of infection [55]. Sore throat, taking vitamin C and smoking remained significant predictors of anxiety levels among participants upon further analysis. Smoking has been associated with adverse COVID-19 prognosis and smokers are at greater risk of developing severe COVID-19 [56–58].

The majority of our study population intended to take the COVID-19 vaccine when available and to vaccinate their children. Participants who reported higher anxiety were more likely to vaccinate, although a relatively large percentage said they would not take the vaccine. This is similar to a recent study in France [59] and a local study showing 12% vaccine hesitancy among the UAE population [60]. Hesitancy about the vaccine was mainly related to safety and political concerns [59, 60]. This highlights the need for governments to publicize the measures taken to ensure vaccine safety.

The psychological impact of COVID-19 on children in the UAE was assessed for the first time in our study. We found high prevalence of parent reported emotional and anxiety problems and when borderline scores were included in the SDQ score, a quarter of children in our study showed parent reported emotional problems. Higher levels of anxiety and emotional problems were found among school age and adolescent age groups which is consistent with earlier reports from Germany, China, Italy, Spain and Ireland [61–64]. COVID-19 adversely affects the mental health of children, particularly those in lower grades. Social isolation, prolonged school closure, challenges with online learning and uncertainty over assessments and examinations all cause mental stress, especially among adolescents [64]. Although we did not interview children directly, we used parent/teacher questionnaires, which were validated against structured diagnostic interviews. Parents and teachers, and especially parents who are teachers, were the best informants of emotional problems in children. Parents who regularly utilized coping strategies with their children reported higher SDQ scores than those who did not. This highlights the need for educating parents about effective coping strategies and mechanisms particularly for nurturing and implementing resilience in children which will assist in overcoming distress and psychological consequences. Further research should measure the effectiveness of these strategies in addressing anxiety disorders in children. We found that higher parental anxiety was a significant predictor of children’s SDQ score, suggesting that parental anxiety might be a unique factor in explaining anxiety disorders in children. This is consistent with research where mental health service utilization among adolescents was associated with parental anxiety and depression [23]. Furthermore, it is uncertain whether these findings demonstrate the likelihood that anxious parents are more likely to report or recognize anxiety problems in their children, or whether children of parents with anxiety disorders have an increased risk of also being anxious [65]. This should be included in future research on the psychological impact of public health emergencies in this population. Further prospective research will be useful in identifying the determinants and characteristics associated with the onset, course and outcome of anxiety and emotional disorders among adults and children.

### Limitations

The use of convenience sampling and its descriptive nature through an online survey may not allow the generalization of results. However, considering the need for a quick method to assess the psychological impact on a population during a rapidly evolving infectious disease outbreak, the online survey proved best [66]. Responses were collected from all over the UAE in addition to countries outside the UAE (due to online and social media use) with a good response rate allowing for some degree of representativeness. The self-reported data in the survey may lead to response biases specifically for reported behavioral changes, coping strategies and measures taken where participants provide socially desirable results. Self-reported levels of anxiety among adults and emotional disorders in children may not be as accurate as those assessed by healthcare professionals. Furthermore, since no single informant can be considered the gold standard of child psychopathology, interviewing children regarding their own symptoms is necessary and several instruments offer developmentally sensitive screening methods to obtain unique information from young children about their mental health problems. These can include pictorial or multimedia self-report screening for mental disorders including anxiety and emotional problems. However, considering the current pandemic, lockdowns, restricted movement and access to participants, this was not possible. Nonetheless, future research should potentially take this into consideration.

## Conclusion

This is the first study to provide information on the psychological impact of COVID-19 on parents and children in the UAE, with association found between parental and child anxiety. Worry and fear are significant predictors of growing anxiety in the UAE. Policymakers should use the findings from this study to develop effective screening methods and interventions to improve mental health, especially for children. These can include more accessible and innovative approaches to mental health programs such as tele-mental health consultations, production and dissemination of creative audio-visual and engaging material related to COVID-19, online schooling, healthy parenting, mental health awareness and coping mechanisms. Such strategies can reduce the psychological impact of COVID-19 in the UAE and other public health emergencies in the future.

## Supplementary Information

**Additional file 1.**

## Data Availability

The datasets used and/or analysed during the current study are available from the corresponding author on reasonable request.

## Abbreviations

COVID-19: Coronavirus Disease 2019
UAE: United Arab Emirates
GAD: Generalized Anxiety Disorder
GAD-7 Scale: Generalized Anxiety Disorder 7 Scale
SDQ: Strengths and Difficulties Questionnaire
WHO: World Health Organization
SARS: Severe Acute Respiratory Syndrome
UK: United Kingdom
IP: Internet Protocol

## References

[1] WHO. Coronavirus disease (COVID-19) outbreak 2020. Available from: http://www.covid19.who.int/. Accessed 12 June 2020.

[2] WHO. Director General's opening remarks at the media briefing on COVID-19, 11th March 2020 2020 [updated 30-3-2020. Available from: http://www.who.int/dg/speeches/detail/who-director-general-s-opening-remarks-at-the-media-briefing-on-covid-19%2D%2D-11-march-2020. Accessed 12 June 2020

[3] Worldometers.info. COVID-19 CORONAVIRUS PANDEMIC. Dover, Delaware, U.S.A. 2020. Available from: http://www.worldometers.info/coronavirus/. Accessed 7 Mar 2021.

[4] Wang C, Pan R, Wan X, Tan Y, Xu L, McIntyre RS, et al. A longitudinal study on the mental health of general population during the COVID-19 epidemic in China. Brain Behav Immunity. 2020;87:40–8. 10.1016/j.bbi.2020.04.028 PMC7153528 32298802

[5] Rubin GJ, Wessely S (2020). The psychological effects of quarantining a city. Br Med J.

[6] Sim K, Huak Chan Y, Chong PN, Chua HC, Wen SS (2010). Psychosocial and coping responses within the community health care setting towards a national outbreak of an infectious disease. J Psychosom Res.

[7] Balkhy HH, Abolfotouh MA, Al-Hathlool RH, Al-Jumah MA (2010). Awareness, attitudes, and practices related to the swine influenza pandemic among the Saudi public. BMC Infect Dis.

[8] Lau JT, Yang X, Tsui HY, Pang E (2004). SARS related preventive and risk behaviours practised by Hong Kong-mainland China cross border travellers during the outbreak of the SARS epidemic in Hong Kong. J Epidemiol Community Health.

[9] Leung GM, Lam TH, Ho LM, Ho SY, Chan BH, Wong IO, Hedley AJ (2003). The impact of community psychological responses on outbreak control for severe acute respiratory syndrome in Hong Kong. J Epidemiol Community Health.

[10] Huang Y, Zhao N (2020). Generalized anxiety disorder, depressive symptoms and sleep quality during COVID-19 outbreak in China: a web-based cross-sectional survey. Psychiatry Res.

[11] Wang C, Pan R, Wan X, Tan Y, Xu L, Ho CS, et al. Immediate Psychological Responses and Associated Factors during the Initial Stage of the 2019 Coronavirus Disease (COVID-19) Epidemic among the General Population in China. Int J Environ Res Public Health. 2020;17(5):1729. 10.3390/ijerph17051729 PMC7084952 32155789

[12] Rodríguez-Rey R, Garrido-Hernansaiz H, Collado S. Psychological Impact and Associated Factors During the Initial Stage of the Coronavirus (COVID-19) Pandemic Among the General Population in Spain. Front Psychol. 2020;11:1540. 10.3389/fpsyg.2020.01540 PMC7325630 32655463

[13] Shevlin M, McBride O, Murphy J, Miller JG, Hartman TK, Levita L, Mason L, Martinez AP, McKay R, Stocks TVA, Bennett KM, Hyland P, Karatzias T, Bentall RP (2020). Anxiety, depression, traumatic stress and COVID-19-related anxiety in the UK general population during the COVID-19 pandemic. BJPsych Open.

[14] Verma S, Mishra A (2020). Depression, anxiety, and stress and socio-demographic correlates among general Indian public during COVID-19. Int J Soc Psychiatry.

[15] Lakhan R, Agrawal A, Sharma M (2020). Prevalence of depression, anxiety, and stress during COVID-19 pandemic. J Neurosci Rural Pract.

[16] de Girolamo G, Cerveri G, Clerici M, Monzani E, Spinogatti F, Starace F, et al. Mental Health in the Coronavirus Disease 2019 Emergency-the Italian response. JAMA Psychiatry 2020;77(9):974–6. 10.1001/jamapsychiatry.2020.1276 32352480

[17] Thomas J, Barbato M, Verlinden M, Gaspar C, Moussa M, Ghorayeb J, Menon A, Figueiras MJ, Arora T, Bentall RP (2020). Psychosocial correlates of depression and anxiety in the United Arab Emirates during the COVID-19 pandemic. Front Psychiatry.

[18] Shekerdemian LS, Mahmood NR, Wolfe KK, Riggs BJ, Ross CE, McKiernan CA (2020). Characteristics and outcomes of children with coronavirus disease 2019 (COVID-19) infection admitted to US and Canadian pediatric intensive care units. JAMA Pediatr.

[19] Stark AM, White AE, Rotter NS, Basu A (2020). Shifting from survival to supporting resilience in children and families in the COVID-19 pandemic: lessons for informing U.S. mental health priorities. Psychol Trauma Theory Res Pract Policy.

[20] Golberstein E, Wen H, Miller BF (2020). Coronavirus disease 2019 (COVID-19) and mental health for children and adolescents. JAMA Pediatr.

[21] Al-Yateem N, Bani Issa W, Rossiter RC, Al-Shujairi A, Radwan H, Awad M (2020). Anxiety related disorders in adolescents in the United Arab Emirates: a population based cross-sectional study. BMC Pediatr.

[22] Cobham VE, McDermott B, Haslam D, Sanders MR (2016). The role of parents, parenting and the family environment in Children's post-disaster mental health. Curr Psychiatry Rep.

[23] Essau CA (2005). Frequency and patterns of mental health services utilization among adolescents with anxiety and depressive disorders. Depress Anxiety.

[24] Jongerden L, Simon E, Bodden DH, Dirksen CD, Bogels SM (2015). Factors associated with the referral of anxious children to mental health care: the influence of family functioning, parenting, parental anxiety and child impairment. Int J Methods Psychiatr Res.

[25] Burstein M, Ginsburg GS, Tein J-Y (2010). Parental anxiety and child symptomatology: an examination of additive and interactive effects of parent psychopathology. [corrected]. J Abnorm Child Psychol.

[26] Cohodes EM, McCauley S, Gee DG. Parental buffering of stress in the time of COVID-19: family-level factors may moderate the association between pandemic-related stress and youth symptomatology. Res Child Adolesc Psychopathol. 2021;16:1–14. 10.1007/s10802-020-00732-6 PMC7885749 33591457

[27] Cusinato M, Iannattone S, Spoto A, Poli M, Moretti C, Gatta M, et al. Stress, Resilience, and Well-Being in Italian Children and Their Parents during the COVID-19 Pandemic. Int J Environ Res Public Health. 2020;17(22):8297. 10.3390/ijerph17228297 PMC7696524 33182661

[28] Boman F, Stafström M, Lundin N, Moghadassi M, Törnhage CJ, Östergren PO (2016). Comparing parent and teacher assessments of mental health in elementary school children. Scand J Public Health.

[29] Goodman R, Ford T, Simmons H, Gatward R, Meltzer H (2003). Using the strengths and difficulties questionnaire (SDQ) to screen for child psychiatric disorders in a community sample. Int Rev Psychiatry.

[30] Aelterman A, Engels N, Van Petegem K, Pierre VJ (2007). The well-being of teachers in Flanders: the importance of a supportive school culture. Educ Stud.

[31] Telles S, Gupta RK, Bhardwaj AK, Singh N, Mishra P, Pal DK, Balkrishna A (2018). Increased mental well-being and reduced state anxiety in teachers after participation in a residential yoga program. Med Sci Monit Basic Res.

[32] EdArabia. Schools in the UAE. 2020. Available from: http://www.edarabia.com/schools/uae. Accessed 11 Mar 2020.

[33] SurveyMonkey. San Mateo. Available from: http://www.surveymonkey.com. Accessed 13 Mar 2020

[34] Al-Rabiaah A, Temsah MH, Al-Eyadhy AA, Hasan GM, Al-Zamil F, Al-Subaie S (2020). Middle East respiratory syndrome-Corona virus (MERS-CoV) associated stress among medical students at a university teaching hospital in Saudi Arabia. J Infect Public Health.

[35] Bults M, Beaujean DJ, Richardus JH, Voeten HA (2015). Perceptions and behavioral responses of the general public during the 2009 influenza a (H1N1) pandemic: a systematic review. Disaster Med Public Health Preparedness.

[36] DeCoster J, Gallucci M, Iselin A-MR (2011). Best practices for using median splits, artificial categorization, and their continuous alternatives. J Experiment Psychopathol.

[37] Leung GM, Ho LM, Chan SK, Ho SY, Bacon-Shone J, Choy RY (2005). Longitudinal assessment of community psychobehavioral responses during and after the 2003 outbreak of severe acute respiratory syndrome in Hong Kong. Clin Infect Dis.

[38] Spitzer RL, Kroenke K, Williams JB, Lowe B (2006). A brief measure for assessing generalized anxiety disorder: the GAD-7. Arch Intern Med.

[39] Kroenke K, Spitzer RL, Williams JB, Monahan PO, Lowe B (2007). Anxiety disorders in primary care: prevalence, impairment, comorbidity, and detection. Ann Intern Med.

[40] Goodman R (1997). The strengths and difficulties questionnaire: a research note. J Child Psychol Psychiatry.

[41] Goodman A, Goodman R (2009). Strengths and difficulties questionnaire as a dimensional measure of child mental health. J Am Acad Child Adolesc Psychiatry.

[42] Silva TBF, Osório FL, Loureiro SR. SDQ: discriminative validity and diagnostic potential. Front Pyschol. 2015;6:811. 10.3389/fpsyg.2015.00811 PMC4462033 26113840

[43] Goodman A. Scoring the Strengths & Difficulties Questionnaire for age 4–17. Available from: http://www.ehcap.co.uk/content/sites/ehcap/uploads/NewsDocuments/236/SDQEnglishUK4-17scoring-1.PDF. Accessed 17 May 2020

[44] Alyahri A, Goodman R (2006). Validation of the Arabic strengths and difficulties questionnaire and the development and well-being assessment. East Mediterr Health J.

[45] Sawaya H, Atoui M, Hamadeh A, Zeinoun P, Nahas Z (2016). Adaptation and initial validation of the patient health questionnaire - 9 (PHQ-9) and the generalized anxiety disorder - 7 questionnaire (GAD-7) in an Arabic speaking Lebanese psychiatric outpatient sample. Psychiatry Res.

[46] SAS Institute Inc. Base SAS® 9.3 Procedures Guide. Cary NSII. 2011.

[47] Lim GY, Tam WW, Lu Y, Ho CS, Zhang MW, Ho RC (2018). Prevalence of depression in the community from 30 countries between 1994 and 2014. Sci Rep.

[48] Löwe B, Decker O, Müller S, Brähler E, Schellberg D, Herzog W, Herzberg PY (2008). Validation and standardization of the generalized anxiety disorder screener (GAD-7) in the general population. Med Care.

[49] McLean CP, Asnaani A, Litz BT, Hofmann SG (2011). Gender differences in anxiety disorders: prevalence, course of illness, comorbidity and burden of illness. J Psychiatr Res.

[50] Borkovec TD, Ray WJ, Stöber J (1998). Worry: a cognitive phenomenon intimately linked to affective, physiological and interpersonal behavioral processes. Cogn Ther Res.

[51] Paulesu E, Sambugaro E, Torti T, Danelli L, Ferri F, Scialfa G, Sberna M, Ruggiero GM, Bottini G, Sassaroli S (2010). Neural correlates of worry in generalized anxiety disorder and in normal controls: a functional MRI study. Psychol Med.

[52] Sun X, So SH, Chan RCK, Chiu CD, Leung PWL (2019). Worry and metacognitions as predictors of the development of anxiety and paranoia. Sci Rep.

[53] Harper CA, Satchell LP, Fido D, Latzman RD. Functional fear predicts public health compliance in the COVID-19 pandemic. Int J Ment Heal Addict. 2020;27:1–14. 10.1007/s11469-020-00281-5 PMC7185265 32346359

[54] Motta Zanin G, Gentile E, Parisi A, Spasiano D. A Preliminary Evaluation of the Public Risk Perception Related to the COVID-19 Health Emergency in Italy. Int J Environ Res Public Health. 2020;17(9):3024 10.3390/ijerph17093024 PMC7246845 32349253

[55] Liao Q, Cowling BJ, Lam WW, Ng DM, Fielding R (2014). Anxiety, worry and cognitive risk estimate in relation to protective behaviors during the 2009 influenza a/H1N1 pandemic in Hong Kong: ten cross-sectional surveys. BMC Infect Dis.

[56] Eisenberg SL, Eisenberg MJ (2020). Smoking cessation during the COVID-19 epidemic. Nicotine Tobacco Res.

[57] Heerfordt C, Heerfordt IM (2020). Has there been an increased interest in smoking cessation during the first months of the COVID-19 pandemic? A Google Trends study. Public Health.

[58] Vardavas CI, Nikitara K (2020). COVID-19 and smoking: a systematic review of the evidence. Tob Induc Dis.

[59] Peretti-Watel P, Seror V, Cortaredona S, Launay O, Raude J, Verger P, et al. The COCONAL group. A future vaccination campaign against COVID-19 at risk of vaccine hesitancy and politicisation. Lancet Infect Dis. 2020;20(7):769–70. 10.1016/S1473-3099(20)30426-6 PMC7239623 32445713

[60] Alsuwaidi AR, Elbarazi I, Al-Hamad S, Aldhaheri R, Sheek-Hussein M, Narchi H. Vaccine hesitancy and its determinants among Arab parents: a cross-sectional survey in the United Arab Emirates. Hum Vaccin Immunother. 2020;16(12):3163–9. 10.1080/21645515.2020.1753439 PMC8641603 32401612

[61] Tang S, Xiang M, Cheung T, Xiang YT (2021). Mental health and its correlates among children and adolescents during COVID-19 school closure: the importance of parent-child discussion. J Affect Disord.

[62] Ravens-Sieberer U, Kaman A, Erhart M, Devine J, Schlack R, Otto C. Impact of the COVID-19 pandemic on quality of life and mental health in children and adolescents in Germany. Eur Child Adolesc Psychiatry. 2021. 10.1007/s00787-021-01726-5. 10.1007/s00787-021-01726-5 PMC7829493 33492480

[63] O’Sullivan K, Clark S, McGrane A, Rock N, Burke L, Boyle N (2021). A qualitative study of child and adolescent mental health during the COVID-19 pandemic in Ireland. Int J Environ Res Public Health.

[64] Orgilés M, Morales A, Delvecchio E, Mazzeschi C, Espada JP. Immediate Psychological Effects of the COVID-19 Quarantine in Youth From Italy and Spain. Front Psychol. 2020;11:579038. 10.3389/fpsyg.2020.579038 PMC7677301 33240167

[65] Beesdo K, Knappe S, Pine DS (2009). Anxiety and anxiety disorders in children and adolescents: developmental issues and implications for DSM-V. Psychiatr Clin North Am.

[66] Geldsetzer P (2020). Use of rapid online surveys to assess People's perceptions during infectious disease outbreaks: a cross-sectional survey on COVID-19. J Med Internet Res.

