# Supplementary material for: The psychological impact of the COVID-19 pandemic on adults and children in the United Arab Emirates: a nationwide cross-sectional study
Source: BMC Psychiatry. 2021 May 3;21:224. doi: 10.1186/s12888-021-03213-2 (PMC8090921; doi:10.1186/s12888-021-03213-2)
Supplement: Supplementary file 1 — Additional file 1. [file 12888_2021_3213_MOESM1_ESM.pdf]

Copy of Levels of anxiety and stress among parents and school teachers during the COVID-19 outbreak

We are a group of researchers from universities across the UAE, and we are evaluating the levels of stress and anxiety associated with the recent corona virus also known as COVID-19 outbreak among parents and school teachers around the world. The findings from this research will further improve our preparedness and awareness campaigns as well as tailor interventions and services to the needs of different communities.

Kindly take a few minutes to answer the questions in the survey below, keeping in mind that all responses will remain confidential and you will not be identified in any way.

At the end of the survey, you can choose to provide (or not to provide) your email address for participation in a lucky draw for Apple AirPods.

Thanking you for your time and efforts. Your participation is highly valued.

For any further information or questions please feel free to contact Prof. Rabih Halwani at rhalwani@sharjah.ac.ae or Dr. Basema Saddik at bsaddik@sharjah.ac.ae.
For any ethical concerns please contact Dr. Suhail AlAmad at salamad@sharjah.ac.ae

Prof. Rabih Halwani, Dr. Basema Saddik, Dr. Ammar AlBanna, Dr. Amal Hussein, Dr. Iffat ElBarazi, Dr. Fatemeh Sharif-Askari.

* 1. **Please indicate your willingness to participate in this survey**

- ○ I am a parent, and I ACCEPT to participate in this survey
- ○ I am a teacher, and I ACCEPT to participate in this survey
- ○ I am neither a parent nor a teacher and I ACCEPT to participate in this survey
- ○ I do NOT ACCEPT to participate in this survey

جامعة الشارقة
UNIVERSITY OF SHARJAH

Copy of Levels of anxiety and stress among parents and school teachers during the COVID-19 outbreak

* 2. What is your gender?

- ◯ Female
- ◯ Male

* 3. What is your age in years?

- ☐ 18-24
- ☐ 25-34
- ☐ 35-44
- ☐ 45-54
- ☐ 55-64
- ☐ 65+

* 4. What is your educational level?

- ☐ Primary
- ☐ Lower secondary
- ☐ Higher secondary
- ☐ Bachelor's degree
- ☐ Post-graduate education

* 5. Where are you located?

- ◯ Inside the UAE
- ◯ Outside the UAE (please specify)

6. Which emirate are you from?

- [ ] Abu Dhabi
- [ ] Ajman
- [ ] Umm Al-Quwain
- [ ] Dubai
- [ ] Ras Al Khaimah
- [ ] I am not in the UAE
- [ ] Sharjah
- [ ] Fujairah
- [ ] Other (please specify)

* 7. What is your employment status?

- [ ] Employed
- [ ] Not employed
- [ ] Home duties

* 8. What is your monthly family income (Dir-hams)/ select other below if different currency)?

- [ ] less than 5000
- [ ] 10,000-19,999
- [ ] more than 40,000
- [ ] 5,000-9,999
- [ ] 20,000-39,000
- [ ] Other (please specify in your local currency)

* 9. Do you have health/medical insurance?

- ( ) No
- ( ) Yes
- ( ) Other (please specify)

* 10. What is your marital status?

- [ ] Single
- [ ] Divorced/separated
- [ ] Married
- [ ] Widowed

* 11. Do you have children?

- ( ) No
- ( ) Yes (How many?)

* 12. What are the ages of your children? (Select all that apply)

- [ ] Infants/toddlers (0 to 3 years)
- [ ] Preschoolers (3 to 5 years)
- [ ] School age (6 to 12 years)
- [ ] Adolescents (13 to 18 years)
- [ ] Young adults (19 to 21 years)
- [ ] I don't have children

* 13. Which level of schooling are they at? (Select all that apply)

- [ ] No school/at home
- [ ] Childcare
- [ ] Primary
- [ ] Lower secondary
- [ ] Other (please specify)
- [ ] Higher secondary
- [ ] University
- [ ] I don't have children

جامعـــــة الشــــــارقة
UNIVERSITY OF SHARJAH

Copy of Levels of anxiety and stress among parents and school teachers during the COVID-19 outbreak

The next few questions will assess your knowledge and beliefs about the recent COVID-19 outbreak

* 14. What do you think about each of the following statements about COVID-19?

	True	False	Don't know
There is no specific treatment	○	○	○
I feel a sense of social responsibility by staying at home	○	○	○

* 15. What do you think of the following statements?

	Very likely	Somewhat likely	Not very likely	Not likely at all	Don't know
There is likelihood of catching COVID-19 during the current outbreak	○	○	○	○	○
There is likelihood of surviving COVID-19 if infected	○	○	○	○	○
There is a likelihood that I develop severe illness if I catch COVID-19	○	○	○	○	○

* 16. Please describe how often you have personally followed the practices below since the recent COVID-19 outbreak

	Always	Most of the time	Occasionally	Never
Covering mouth when coughing or sneezing	◯	◯	◯	◯
Using hand sanitizer more frequently	◯	◯	◯	◯
Washing hands with soap	◯	◯	◯	◯
Wearing face mask	◯	◯	◯	◯
Avoiding crowded areas (e.g. malls)	◯	◯	◯	◯
Avoiding public transport	◯	◯	◯	◯
Avoiding handshakes/personal contact with others	◯	◯	◯	◯

جامعة الشارقة
UNIVERSITY OF SHARJAH

Copy of Levels of anxiety and stress among parents and school teachers during the COVID-19 outbreak

The next FEW questions will assess your levels of anxiety and worry in regards to the recent Corona outbreak. Please take your time considering your most suitable choices.

* 17. Since the COVID-19 outbreak, how often have you been bothered by the following problems?

	Not at all	Several days	More than half the days	Nearly every day
Feeling nervous, anxious or on edge	○	○	○	○
Not being able to stop or control worrying	○	○	○	○
Worrying too much about different things	○	○	○	○
Trouble relaxing	○	○	○	○
Being so restless that it is hard to sit still	○	○	○	○
Becoming easily annoyed or irritable	○	○	○	○
Feeling afraid as if something awful might happen	○	○	○	○

* 18. If you checked off any problems above, how difficult have these problems made it for you to do your work, take care of things at home, or get along with other people?

Not difficult at all	Somewhat difficult	Very difficult	Extremely difficult
○	○	○	○

* 19. I feel less anxious with the introduction of the following precautionary measures

	Strongly agree	Agree	Neither agree nor disagree	Disagree	Strongly disagree
Online learning at educational institutions	◯	◯	◯	◯	◯
Airport screening	◯	◯	◯	◯	◯
Travel bans	◯	◯	◯	◯	◯
Availability of hand sanitizers in public places (e.g. malls, banks, restaurants)	◯	◯	◯	◯	◯
Cancellation of social events (e.g. sports, concerts, social gatherings)	◯	◯	◯	◯	◯
Temporary closure of public places (e.g. gyms, museums, parks)	◯	◯	◯	◯	◯
Social isolation	◯	◯	◯	◯	◯

* 20. What is your opinion about the following statements describing the public fear that is associated with the recent COVID-19 outbreak?

	Strongly agree	Agree	Neither agree nor disagree	Disagree	Strongly disagree
The public fear is justifiable/ appropriate	◯	◯	◯	◯	◯
The fear is dysfunctional (has caused unnecessary absence from school or work)	◯	◯	◯	◯	◯

* 21. How worried have you felt over the past few weeks about the following?

	Extremely worried	Very worried	Somewhat worried	Little worried	Not worried at all	N/A
I am worried about catching COVID-19 myself	◯	◯	◯	◯	◯	◯
I am worried about one of my parents catching COVID-19	◯	◯	◯	◯	◯	◯
I am worried about one of my children catching COVID-19	◯	◯	◯	◯	◯	◯
I am worried about what COVID-19 can do to me health-wise	◯	◯	◯	◯	◯	◯
I am worried about social isolation/quarantine	◯	◯	◯	◯	◯	◯
I am worried about the loss of income if infected with COVID-19	◯	◯	◯	◯	◯	◯
I am worried about transmitting COVID-19 from my workplace to my family members/friends	◯	◯	◯	◯	◯	◯

* 22. For the next question, it would help us if you answered all items as best you can even if you are not absolutely certain.

Since the COVID-19 outbreak, I have noticed the following changes in my child/ren's behavior.

	Not true	Somewhat true	Certainly true	N/A
Often complains of headaches, stomach aches or sickness	◯	◯	◯	◯
Has many worries, often seems worried	◯	◯	◯	◯
Often unhappy, down-hearted or tearful	◯	◯	◯	◯
Nervous or clingy in new situations, easily loses confidence	◯	◯	◯	◯
Has many fears, easily scared	◯	◯	◯	◯

* 23. If you answered "somewhat true" or "certainly true" to any of the previous questions, how many of your children displayed these behaviours?

- [ ] 1
- [ ] 2
- [ ] 3
- [ ] 4
- [ ] 5+
- [ ] Not Applicable

* 24. Which of the following practices have you undertaken to help reduce anxiety in your children/family during the COVID-19 outbreak?

	Always	Most of the time	Occasionally	Never	N/A
I have openly discussed the COVID-19 with my children/family	◯	◯	◯	◯	◯
I have educated my child/ren about proper protective measures (e.g. hand hygiene)	◯	◯	◯	◯	◯
I have reassured my child/ren that they are safe	◯	◯	◯	◯	◯
I have limited my child/ren's exposure to news coverage of COVID-19, including social media	◯	◯	◯	◯	◯
I have created a schedule for learning and fun activities	◯	◯	◯	◯	◯
I have tried to maintain a regular routine at home (e.g. dinner, bedtime)	◯	◯	◯	◯	◯

Copy of Levels of anxiety and stress among parents and school teachers during the COVID-19 outbreak

* 25. If a vaccine is developed for COVID-19 in the near future, how likely are you:

	Very likely	Somewhat likely	Not very likely	Not likely at all	I don't know	N/A
To take the vaccine yourself?	○	○	○	○	○	○
To vaccinate your children?	○	○	○	○	○	○

* 26. Do you suffer from any of the following pre-existing medical conditions? (Select all that apply)

- ☐ Diabetes Mellitus
- ☐ Heart problems
- ☐ High blood pressure
- ☐ Dyslipidemia
- ☐ Other (please specify)
- ☐ Asthma
- ☐ Respiratory problems
- ☐ Cancer
- ☐ None of the above

* 27. Have you or your family members experienced any of the following symptoms over the last 2 weeks? (Select all that apply)

- [ ] Headaches
- [ ] Fever
- [ ] Cough
- [ ] Difficulty breathing
- [ ] Sore throat
- [ ] Other (please specify)
- [ ] Myalgia
- [ ] Dizziness
- [ ] Runny nose
- [ ] Diarrhea
- [ ] None of the above

* 28. What measures have you taken over the last few weeks for any flu like symptoms? (Select all that apply)

- [ ] Vitamin C
- [ ] Flu medications (e.g. Tamiflu)
- [ ] Anti-inflammatory drugs (e.g. Aspirin, Ibuprofen (Advil, Nurofen))
- [ ] Analgesics and anti-pyretics (e.g. Paracetamol, Tylenol, Panadol)
- [ ] Other (please specify)
- [ ] Oral Steroids (e.g. Corticosteroids, cortisone, prednisone)
- [ ] Herbal remedies
- [ ] No measures taken

* 29. My child/ren are up to date with their vaccinations

- No
- Yes
- I don't have children

* 30. Do you smoke?

- No
- Yes
- I used to smoke but I have now quit smoking

جامعـــــــة الشـــــــارقة
UNIVERSITY OF SHARJAH

Copy of Levels of anxiety and stress among parents and school teachers during the COVID-19 outbreak

* 31. What do you smoke? (Select all that apply)

- [ ] Cigarettes
- [ ] Shisha
- [ ] Midwakh
- [ ] Vaping
- [ ] Other (please specify)

* 32. Have your smoking habits changed since the COVID-19 outbreak?

- ( ) No
- ( ) Yes (please specify what changes you have made)

33.

Thank you for completing this survey. We appreciate your time and efforts. If you would like to participate in a lucky draw for Apple AirPods, please provide your email address below.
